# Cost analysis of voriconazole versus liposomal amphotericin B for primary therapy of invasive aspergillosis among patients with haematological disorders in Germany and Spain

**DOI:** 10.1186/2050-6511-15-52

**Published:** 2014-09-24

**Authors:** Helmut Ostermann, Carlos Solano, Isidro Jarque, Carolina Garcia-Vidal, Xin Gao, Jon Andoni Barrueta, Marina De Salas-Cansado, Jennifer Stephens, Mei Xue, Bertram Weber, Claudie Charbonneau

**Affiliations:** 1 Medical Clinic III, Department of Haematology and Oncology, University Hospital Munich – Grosshadern, Munich, Germany; 2 Haematology and Oncology Department, Hospital Clínico Universitario INCLIVA, University of Valencia, Valencia, Spain; 3 Haematology Service, Hospital Universitari i Politècnic La Fe, Valencia, Spain; 4 Hospital Universitari de Bellvitge, Universitat de Barcelona, Barcelona, Spain; 5 Pharmerit International, Bethesda, MD, USA; 6 Medical Unit, Pfizer Spain, Madrid, Spain; 7 Health Economics and Outcomes Research Department, Pfizer Spain, Madrid, Spain; 8 Health Technology Assessment and Outcomes Research Department, Pfizer Germany, Berlin, Germany; 9 Health Economics and Outcomes Research Department, Pfizer International Operations, Paris, France

**Keywords:** Aspergillosis, Cost-effectiveness, Decision-analytic model, Invasive fungal disease, Pharmacoeconomics

## Abstract

**Background:**

The current healthcare climate demands pharmacoeconomic evaluations for different treatment strategies incorporating drug acquisition costs, costs incurred for hospitalisation, drug administration and preparation, diagnostic and laboratory testing and drug-related adverse events (AEs). Here we evaluate the pharmacoeconomics of voriconazole versus liposomal amphotericin B as first-line therapies for invasive aspergillosis (IA) in patients with haematological malignancy and prolonged neutropenia or who were undergoing haematopoietic stem-cell transplantation in Germany or Spain.

**Methods:**

A decision analytic model based on a decision tree was constructed to estimate the potential treatment costs of voriconazole versus liposomal amphotericin B. Each model pathway was defined by the probability of an event occurring and the costs of clinical outcomes. Outcome probabilities and cost inputs were derived from the published literature, clinical trials, expert panels and local database costs. In the base case, patients who failed to respond to first-line therapy were assumed to experience a single switch between comparator drugs or the other drug was added as second-line treatment. Base-case evaluation included only drug-management costs and additional hospitalisation costs due to severe AEs associated with first- and second-line therapies. Sensitivity analyses were conducted to assess the robustness of the results. Cost estimates were inflated to 2011 euros (€).

**Results:**

Based on clinical trial success rates of 52.8% (voriconazole) and 50.0% (liposomal amphotericin B), voriconazole had lower total treatment costs compared with liposomal amphotericin B in both Germany (€12,256 versus €18,133; length of therapy [LOT] = 10-day intravenous [IV] + 5-day oral voriconazole and 15-day IV liposomal amphotericin B) and Spain (€8,032 versus €10,516; LOT = 7-day IV + 8-day oral voriconazole and 15-day IV liposomal amphotericin B). Assuming the same efficacy (50.0%) in first-line therapy, voriconazole maintained a lower total treatment cost compared with liposomal amphotericin B. Cost savings were primarily due to the lower drug acquisition costs and shorter IV LOT associated with voriconazole. Sensitivity analyses showed that the results were sensitive to drug price, particularly the cost of liposomal amphotericin B.

**Conclusions:**

Voriconazole is likely to be cost-saving compared with liposomal amphotericin B when used as a first-line treatment for IA in Germany and Spain.

## Background

Invasive fungal diseases (IFDs) are a significant cause of morbidity and mortality in immunocompromised patients, and are associated with increased healthcare costs [1]. The frequency of IFDs has increased substantially over recent years, largely due to the increasing size of the population at risk, which includes transplant recipients, patients with haematological malignancies, and patients with HIV [2].

Invasive aspergillosis (IA) is the most significant IFD in immunocompromised patients, with an incidence rate of approximately 10% in allogeneic bone marrow transplant recipients [3], and an overall case-fatality rate of 58% (range 43–87% depending on underlying disease) [4]. As a result, patients with IA have a significantly increased length of stay (LOS) in hospital and increased healthcare costs compared with patients without IA [5-8]. The direct costs associated with IA are substantial and include inpatient and outpatient costs, such as increased LOS in hospital, costs of antifungal therapy, and costs related to the treatment of drug-related adverse events (AEs) [9].

Effective treatment of IFD is an ongoing challenge to the clinical community from both a clinical and cost perspective. Across Europe, treatment recommendations are managed at a national level and are based on a range of factors including: clinical outcomes (e.g. infection resolution and mortality), economic outcomes (e.g. treatment costs and hospital LOS) and quality of life information. In Germany, the Infectious Diseases Working Party of the German Society of Haematology and Oncology provides guidelines for the diagnosis [10] and treatment [11] of IFD in patients with haemato-oncological disorders. In Spain, the Third European Conference on Infections and Leukaemia [12] and the Spanish Society of Infectious Diseases and Clinical Microbiology [13] provide specific therapies and disease-management strategies.

Voriconazole (Vfend®, Pfizer Inc) and liposomal amphotericin B (Ambisome®, Gilead Sciences) are licensed for the treatment of IFD, including IA. In a prospective, randomised trial of voriconazole versus amphotericin B deoxycholate for the primary treatment of IA, voriconazole demonstrated superior efficacy and improved survival, and resulted in fewer side effects, compared with amphotericin B [14].

Health economic-based models are designed to address questions of economic relevance, and integrate efficacy and safety data from clinical trials with medical resource use and quality of life information from the published literature, expert opinion and database analysis. Here, we evaluate the pharmacoeconomics of voriconazole versus liposomal amphotericin B as first-line therapies for IA in patients with haematological malignancy and prolonged neutropenia, or those undergoing haematopoietic stem cell transplantation (HSCT), from German and Spanish hospital perspectives.

## Methods

### Study design

**Figure 1:**
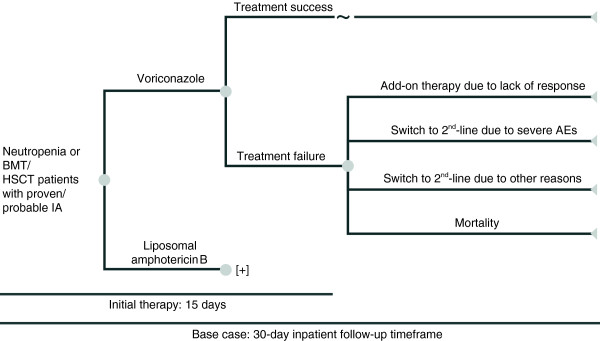
**Decision analytic model.** AE, adverse event; BMT, bone marrow transplant; HSCT, haematopoietic stem-cell transplant; IA, invasive aspergillosis.

This analysis was conducted from German and Spanish hospital perspectives. The study population comprised patients with haematological malignancy and prolonged neutropenia or those undergoing HSCT. Eligible patients met the criteria for proven or probable IA defined in the key randomised clinical trials of voriconazole [14] and liposomal amphotericin B [15]. Briefly, the key voriconazole trial enrolled immunocompromised patients aged ≥12 years with definite or probable IA. Patients were excluded if they had chronic aspergillosis, aspergilloma or allergic bronchopulmonary aspergillosis [14]. The key liposomal amphotericin B trial enrolled patients who met the criteria for proven or probable invasive mould infections as established by the European Organisation for Research and Treatment of Cancer/Mycosis Study Group; in addition, a protocol-defined modification allowed a diagnosis of probable IA in patients with a halo or air crescent sign on chest computed tomography scan who had undergone allogeneic stem cell transplantation or who had neutropenia within 14 days of study entry [15].To estimate the potential therapy costs of voriconazole versus liposomal amphotericin B, a decision analytic model based on a decision tree was developed (Figure 1). Each pathway in the model was defined by the probability of an event occurring and the costs associated with each clinical outcome defined as: (1) treatment success or (2) treatment failure, which included add-on treatment due to lack of response, switching to second-line therapy due to serious AEs or for other reasons, and mortality.

The model inputs were treatment options, length of therapy (LOT), additional LOS in hospital, other efficacy and safety inputs, and drug costs. Outcome measures were the total hospital costs (e.g. drug and hospitalisation costs) associated with each therapy in 2011 euros (€). Outcome probabilities and cost inputs were derived from the published literature, clinical trials, expert panel discussions and local database costs.

### Key model assumptions

The model time horizon was the 30-day inpatient follow-up period based on the mean LOS for HSCT patients with IA and haematological malignancy in a United States claims database analysis [16].

In the base-case scenario, patients who failed to respond to first-line therapy were assumed to undergo a single switch between comparator drugs or the other drug was added as a second-line therapy. Drug dosages were based on patients having an average weight of 70 kg, as defined in the key randomised clinical trials of voriconazole [14] and liposomal amphotericin B [15]. Voriconazole was administered intravenously at doses of 6 mg/kg twice daily (BID; Day 1) and 4 mg/kg BID (Day 2+), and orally at a dose of 200 mg BID. Intravenous (IV) liposomal amphotericin B was administered at a dose of 3 mg/kg once daily*.* It was assumed that there was no vial wastage during use.

The first-line drugs, voriconazole and liposomal amphotericin B, were only used in an inpatient setting (mean LOS = 30 days). Second-line therapies could be used in both inpatient and outpatient settings. IV LOT was determined by clinical experts from each country. In the German base-case scenario, first-line LOT was 10 days of IV voriconazole followed by 5 days of oral voriconazole and 15 days of IV liposomal amphotericin B. Second-line LOT was 15 days of IV voriconazole, liposomal amphotericin B or caspofungin. In the Spanish base-case scenario, first-line LOT was 7 days of IV voriconazole followed by 8 days of oral voriconazole and 15 days of IV liposomal amphotericin B. Second-line LOT was 7 days of IV voriconazole followed by 8 days of oral voriconazole and 15 days of IV liposomal amphotericin B or caspofungin.

After the initial success of IV voriconazole, conversion to oral voriconazole could be used as a first-line therapy, and it could also be used as a second-line add-on or switch-to therapy. If the comparator drug was added as second-line combination therapy due to lack of response with the first-line monotherapy, the first-line drug (voriconazole or liposomal amphotericin B) was continued as IV therapy (liposomal amphotericin B) or as a combination of IV and oral therapy (voriconazole) for the same duration as the add-on drug.

### Cost calculation assumptions

The base-case evaluation included only drug-management costs and additional hospitalisation costs due to severe AEs associated with first- and second-line therapies.

Based on the results of the key randomised clinical trials, severe AEs with first- and second-line therapies included nephrotoxicity and hypokalaemia [14,15]. Although the effects of infusion-related reactions (e.g. visual disturbances, chest pain and back pain) were reported to be significantly different between the two treatment arms, it was assumed that the impact of moderate/severe infusion-related reactions would be captured by the pathway of treatment failure/switch due to AEs, and that additional costs incurred due to mild infusion-related reactions were minimal.

Hospital LOS was not included in the cost calculation as it was assumed to be similar across the different therapies. Based on a previously published study that assessed additional resource utilisation associated with AEs, an additional 2.2 days of hospital stay was applied to both treatment arms in the base-case scenario to reflect the impact of severe AEs [17].

### Clinical input

Clinical efficacy, safety and mortality data in this model were based on the default drug dosages and were obtained from the key randomised clinical trials of voriconazole [14] and liposomal amphotericin B [15] (Table 1). The probability of adding another antifungal drug to the regimen due to lack of response was calculated as 1 minus the switch rate to second-line therapy.

**Table 1:** Efficacy and safety input data

**Treatment**	**Treatment success rate (%)**	**Add-on rate (%)**	**Severe AEs/switch due to severe AEs (%)**	**Switch (due to other reason) (%)**	**AE rate (%)**	**Mortality rate (%)**
Voriconazole	52.8	19.2	13.4	9.0	13.4	5.6
Liposomal amphotericin B	50.0	8.7	20.0	16.0	20.0	5.3

Data sources: [14,15].

AE, adverse event.

### Costs of medical resource and drug acquisition

Hospitalisation costs and outpatient IV administration costs were based on economic research on the treatment of IA in Germany [1,18,19] and Spain [20] (Table 2). Drug acquisition costs in Germany were derived from the LAUER-Taxe, Pharmindex for Germany drug costs (Table 3) [21]. Drug acquisition costs in Spain were derived from the Bot Plus database (Table 3) [22].

**Table 2:** Inpatient and outpatient costs (2011 €)

		**Price (€)**	
**Setting**	**Description**	**Germany**	**Spain**
Inpatient	Hospitalisation cost per day	674.10	566.52
Outpatient	Intravenous administration cost per unit	34.90	34.90

Data sources: [1,18-20].

**Table 3:** Drug acquisition costs (2011 €)

		**Unit price (per vial or tablet) (€)**	
**Treatment**	**Dosage**	**Germany**	**Spain**
Voriconazole	200 mg IV	156.00	133.32
	Oral tablet 200 mg	45.00	35.68
Liposomal amphotericin B	50 mg IV	220.47	130.06
Caspofungin	70 mg IV	793.24	570.81
	50 mg IV	625.70	448.76

Data sources: [21,22].

IV, intravenous.

### Sensitivity analysis

The effects of individual parameters (e.g. drug price and hospital costs) were examined by varying estimates by ±30%. Sensitivity results for each input were ranked from the most sensitive to the least sensitive via a tornado diagram.

## Results

### German hospital perspective

**Figure 2:**
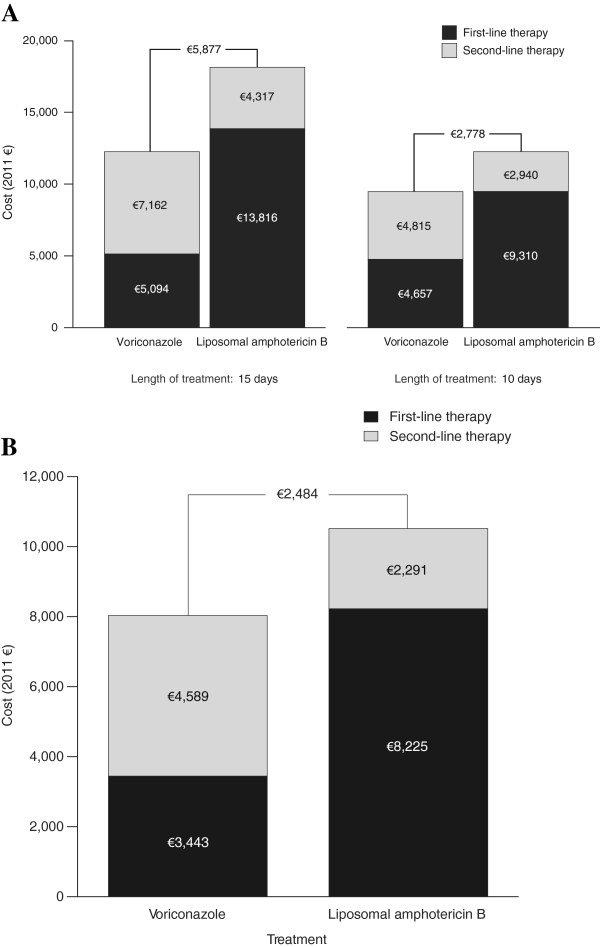
Cost comparison for base-case scenario in (A) Germany and (B) Spain.

**Figure 3:**
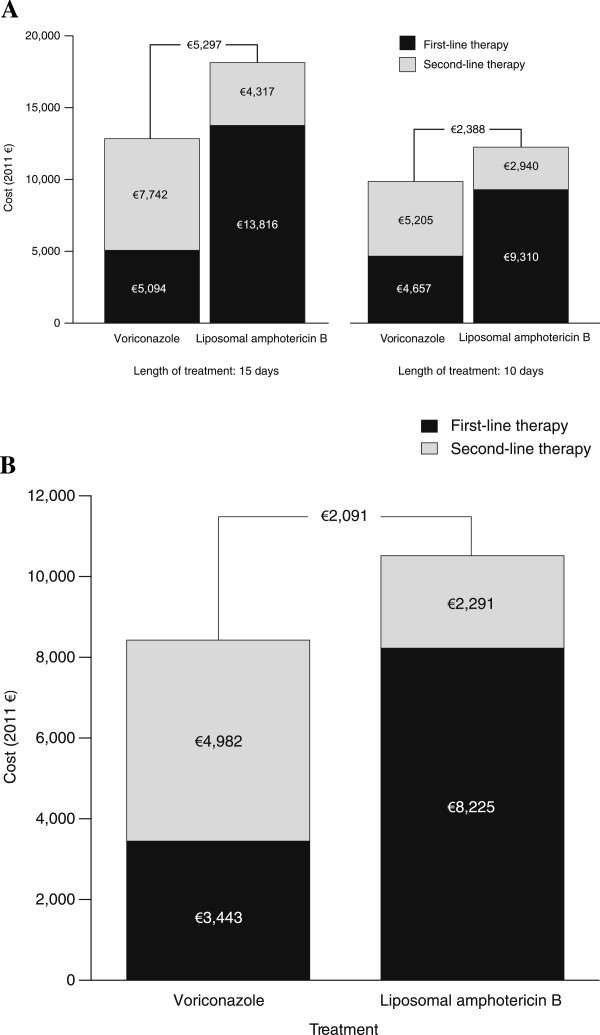
Cost comparison assuming equal efficacy in (A) Germany and (B) Spain.

Based on clinical trial success rates of 52.8% for voriconazole and 50.0% for liposomal amphotericin B and a LOT of 10 days for IV voriconazole followed by 5 days for oral voriconazole and 15 days for IV liposomal amphotericin B, voriconazole had a lower total treatment cost compared with liposomal amphotericin B (€12,256 versus €18,133) (Figure 2A). If the same LOT of 10 days was assumed for both arms (IV only), voriconazole maintained a lower total treatment cost of €9,472 compared with the total treatment cost of €12,250 for liposomal amphotericin B. If the same efficacy (50.0%) was assumed for both first-line therapies, voriconazole maintained a lower total treatment cost compared with liposomal amphotericin B (€12,836 versus €18,133, respectively, over 15 days of treatment, and €9,862 versus €12,250, respectively, over 10 days of treatment) (Figure 3A).

When total treatment costs were broken down by line of therapy, first-line therapy costs were substantially lower for voriconazole versus liposomal amphotericin B, whereas second-line therapy costs were lower for liposomal amphotericin B versus voriconazole. These observations remained consistent regardless of whether LOT was 10 or 15 days and whether efficacy assumptions were based on clinical trial success rates or equivalency. AEs accounted for 3.9% (€199 of €5,094) and 2.1% (€297 of €13,816) of the total costs of first-line treatment with voriconazole and liposomal amphotericin B, respectively.

The cost savings associated with voriconazole were primarily due to lower drug acquisition costs and a shorter IV LOT combined with the use of a cheaper oral formulation. AE savings accounted for 1.0% of the total cost saving associated with first-line voriconazole versus liposomal amphotericin B.

### Spanish hospital perspective

Based on clinical trial success rates of 52.8% for voriconazole and 50.0% for liposomal amphotericin B and a LOT of 7 days for IV voriconazole followed by 8 days of oral voriconazole and 15 days for IV liposomal amphotericin B, voriconazole had a lower total treatment cost compared with liposomal amphotericin B (€8,032 versus €10,516) (Figure 2B). When the same efficacy of 50.0% was assumed for both first-line therapies, voriconazole maintained a lower total treatment cost compared with liposomal amphotericin B (€8,425 versus €10,516, respectively) (Figure 3B).

Similar to the German hospital perspective, when total treatment costs were broken down by therapy line, first-line therapy costs were substantially lower for voriconazole versus liposomal amphotericin B, whereas second-line therapy costs were lower for liposomal amphotericin B versus voriconazole. AEs accounted for 4.9% (€167 of €3,443) and 3.0% (€249 of €8,225) of the total costs of first-line treatment with voriconazole and liposomal amphotericin B, respectively.

Cost savings with voriconazole were primarily due to lower drug acquisition costs and a shorter IV LOT combined with the use of a cheaper oral formulation. AE savings accounted for 2.0% of the total cost saving associated with first-line voriconazole versus liposomal amphotericin B.

### Sensitivity analyses

Sensitivity analyses showed that the results of the model were sensitive to drug price, particularly the cost of liposomal amphotericin B. In the German hospital scenario (LOT = 15 days) a variation in drug price of ±30% resulted in upper and lower bounds for the €5,878 total cost saving of €8,561 and €3,194 for liposomal amphotericin B, €5,070 and €6,685 for the IV formulation of voriconazole, and €5,746 and €6,009 for the oral formulation of voriconazole. Similarly, in the Spanish hospital scenario (LOT = 15 days), a variation in drug price of ±30% resulted in upper and lower bounds for the €2,483 total cost saving of €4,066 and €900 for liposomal amphotericin B, €1,719 and €3,247 for the IV formulation of voriconazole, and €2,393 and €2,573 for the oral formulation of voriconazole.

## Discussion

Pharmacoeconomic analyses are increasingly important in the clinical arena where decision-makers face growing pressure to optimise value and quality of care. In addition, pharmacoeconomic analyses can also provide information to support clinical decision-making to promote the most effective and appropriate treatment for patients. Improvements in efficacy and reductions in hospital LOS and costs associated with AEs are clearly also desirable outcomes for patients and clinicians.

The pharmacoeconomic evaluation described in this article applies to the treatment of IA infections in patients with haematological disorders in Germany and Spain. The findings from our model, together with the previously reported results from a randomised clinical trial [14], suggest that voriconazole is likely to be cost-saving as first-line therapy compared with liposomal amphotericin B, and is a better treatment option from a clinical, safety and economic perspective. This observation was consistent across all scenarios tested in this analysis.

To the best of our knowledge, this is the first analysis to directly compare the pharmacoeconomic costs of voriconazole and liposomal amphotericin B for the treatment of IA. Wingard and colleagues compared the resource use and cost of treatment for voriconazole with conventional amphotericin B for IA and found that using voriconazole as a first-line therapy resulted in significantly fewer deaths and similar treatment costs [23]. Surviving patients who were treated with voriconazole spent fewer days in intensive care and more days out of hospital than those who received amphotericin B [23]. In addition, decision-tree modelling has suggested that voriconazole is cost-saving overall compared with conventional amphotericin B in clinical trial populations in the United States, Canada, Germany and the Netherlands [18,24-26].

Cost savings associated with voriconazole were thought to be attributable to its lower drug acquisition costs and shorter IV LOT due to the availability of an oral formulation of voriconazole. In the German model, a reduction in the IV LOT from 15 to 10 days had a great impact on the total treatment cost for liposomal amphotericin B, reducing the total cost by approximately one-third. In clinical practice, the IV LOT for voriconazole is often shorter than 10 days, which could lead to even greater cost savings. In addition to cost savings, the availability of an oral formulation of voriconazole offers several other advantages, including ease of delivery, reduced infection risk in an already immunocompromised patient population, and improved patient compliance.

The limitations of this study included the use of clinical trial data that may not be representative of the general population, the absence of head-to-head data comparing voriconazole with liposomal amphotericin B, and the assumption of 100% efficacy with second-line therapy. However, this assumption was required to prevent extrapolation of the time horizon beyond 30 days and was considered acceptable by clinical experts as no large trials evaluating efficacy data for second-line therapies have been conducted in this patient population.

Several factors, including drug-drug interactions and AEs, contribute to the long-term costs of IA. As voriconazole is an inhibitor of CYP3A4 liver enzymes, its concurrent use with other agents that are metabolized by the same system may result in substantial drug-drug interactions (e.g., concomitant use with immunosuppressive transplant medications) [27-29]. Voriconazole has also been associated with an increased risk of hepatotoxicity, with studies reporting significant transaminase abnormalities in 12.4% of patients [30]; however, most instances of drug-induced hepatotoxicity are unpredictable and vary considerably depending on the population, indication, formulation, and dosage [31,32]. Although liposomal amphotericin B has been shown to be substantially less toxic than conventional amphotericin B, particularly with respect to infusion-related reactions and nephrotoxicity, its use is still limited by these AEs [33]. Furthermore, concurrent use of liposomal amphotericin B with other nephrotoxic agents (e.g., cyclosporine, aminoglycosides, polymixins, tacrolimus and pentamidine) has been shown to increase the potential for drug-induced nephrotoxicity in some patients [34].

## Conclusions

This study showed that voriconazole is likely to be cost-saving compared with liposomal amphotericin B for the first-line treatment of IA infections in patients with haematological disorders in Germany and Spain. These conclusions are valid for countries with a comparable healthcare system and comparable drug costs; however, studies using observational real-world data are required to confirm these findings.

## Abbreviations

AE: Adverse event; BID: Twice daily; HSCT: Haematopoietic stem cell transplantation; IA: Invasive aspergillosis; IFD: Invasive fungal disease; IV: Intravenous; LOS: Length of stay; LOT: Length of therapy.

## Competing interests

Helmut Ostermann has received research grants from Gilead Sciences and MSD; has received compensation for attending advisory boards for Astellas Pharma, Gilead Sciences, and MSD; and has served on the speaker’s bureau for Astellas Pharma, Gilead Sciences, MSD, and Pfizer Inc. Carlos Solano has served as a consultant for Astellas Pharma, Gilead Sciences, MSD, and Pfizer Inc; has received research grants from Astellas Pharma, Gilead Sciences, Pfizer Inc and the Instituto de Salud Carlos III (Spanish Ministry of Economy and Competitiveness); and has received honoraria for talks from Astellas Pharma, Gilead Sciences, MSD, and Pfizer Inc. Isidro Jarque has served as a consultant for Astellas Pharma, Gilead Sciences, MSD, and Pfizer Inc. Carolina Garcia-Vidal has no competing interests. Jon Andoni Barrueta, Marina De Salas-Cansado, Bertram Weber, and Claudie Charbonneau are employees of Pfizer Inc. Xin Gao, Jennifer Stephens, and Mei Xue are employees of Pharmerit International, and have served as consultants for Pfizer Inc.

## Authors’ contributions

XG and JS contributed to model conceptualisation, design, and assumptions. XG and MX conducted the model programming and analyses. All authors contributed to the review and interpretation of the results, and the development and critical review of the manuscript. All authors read and approved the final manuscript.

## Pre-publication history

The pre-publication history for this paper can be accessed here:

http://www.biomedcentral.com/2050-6511/15/52/prepub
